# Case of Inversio-Uteri

**Published:** 1856-06

**Authors:** Samuel P. Brown

**Affiliations:** Greensburgh, Pa.


### MEDICAL EXAMINER.

NEW SERIES.—NO. CXXXVIII.—JUNE, 1856.

### ORIGINAL COMMUNICATIONS.

### *Case of Inversio-Uteri.* By Samuel P. Brown, M. D., of Greensburgh, Pa.

Mrs. E., a healthy young woman, in her second confinement, requested my attendance on the morning of the 3d of April, 1856, at 3 o'clock, A. M. I found she had been in labor all night, the pains recurring at intervals of ten and fifteen minutes, short and without tenesmus; this continued until about 8 o'clock, A. M., when they became more frequent and effective. Whilst walking about the room, a strong bearing down pain came on, during which she got on her knees close by the bed; I went to her assistance, when, with a violent expulsive effort, the fœtus was extruded. She immediately complained of exhaustion and pain in the abdomen, and desired to be put to bed, which I requested the attendants to do, my attention being directed to the child. In a few moments one of the women called to me that she was flooding profusely. I immediately went to her, and whilst my hand was on the abdomen, she was seized with a violent pain, which I thought expelled the placenta; but what was my astonishment, on introducing my hand under the covering, to find the uterus inverted, with the placenta adherent. Feeling very much alarmed for the safety of my patient, and knowing that my friend A. T. King, M. D., and my son R. Brown, M. D., were about that hour to meet in the neighborhood for the purpose of making a *post mortem* examination, I requested their attendance; meanwhile I attempted to return the uterus with the placenta, but failed. My son arriving at this time, took hold of the uterus and peeled off the placenta, then grasping the part with his hand, re-introduced it into the vagina, carried it through the os uteri, and indenting the fundus, pushed it forward with the fingers in a conical shape, and thus, without much difficulty, succeeded in replacing it. Strong uterine contractions occurred soon afterwards. Dr. King now came in, and at our request made an examination, and found the fundus again slightly depressed.

Under the careful administration of stimulants and anodynes she soon rallied. No hæmorrhage occurred afterwards, and with the exception of retention of urine, which required the use of the catheter twice daily, for the remainder of the week, she had a good getting up. The secretion of milk was not fully established until after the ninth day.

I have been actively engaged in the practice of medicine for upward of thirty years, have attended over three thousand labors, and never met with a case of inversion before. Authors inform us that the causes are atony of the uterus, or active contraction of the one part with atonic condition of another, too violent traction of the cord in extraction of the placenta, want of length in the funis or its shortening by being coiled around the neck of the child, &c. In the case now stated, my opinion is, that depression took place immediately on the birth of the child, from the violent expulsive effort combined with the shortness of the funis, hence the sinking and pain, followed by hæmorrhage, which ceased immediately when the inversion became complete.

[A very interesting case of inversion of the uterus, with reposition on the eighth day, is reported by Prof. White, in the March number of the Buffalo Medical Journal. The patient had been attended by a German midwife, who stated that after a brief labor, she had given birth to a male infant, weighing upwards of 10 pounds. The placenta very soon came away, accompanied by the inverted uterus, which descended into the vagina. The flooding at this time was described as terrific, and produced protracted syncope. No medical assistance was requested for several days afterwards, by which time the tumor had descended through the os externum, and had become suspended between the patient's thighs. The first attempt to replace it by Dr. White proved unsuccessful, in consequence of the fainting of the patient from loss of blood during the effort; the vulva was very sensitive also, and it was therefore deemed prudent to cease all efforts until the following day. On this day, the eighth after labor, her vulvar soreness being much relieved by the fomentations which had been applied, and her strength sustained by the administration of broths and stimulants, although still greatly prostrated, Dr. White again attempted, and at last succeeded, in replacing the inversion. Chloroform, it should be mentioned, was administered during the whole operation. As an instructive example of the plan of proceeding to be adopted under such circumstances, we copy Dr. White's statement in detail:—

"I now placed myself upon my knees between the inferior extremities of the patient, a position admitting of free motion on my own part, giving complete control of the pelvis of the woman, and which could be maintained for a considerable period without unnecessary fatigue to the operator. Introducing the entire right hand into the vagina, the whole body and fundus of the organ were firmly and continuously compressed for some time. At length, keeping up the pressure, it was found, upon applying the thumb to the fundus, that a slight depression could be made. Having succeeded in dimpling the fundus, pressure was maintained with the thumb at that point until the hand became so fatigued as to be nearly powerless. To preserve this depression whilst the muscles of the hand were permitted to relax, a rectum bougie, about twelve inches in length and one in diameter, was carried along its palm fixed in the the dimple, and pressure unintermittingly continued through it by the left hand outside the vulva. So soon as the intro-vaginal hand wa sufficiently rested, pressure by it was recommenced and the bougie withdrawn.

Whenever these progressive efforts were resumed, the left hand was placed over the uterine tumor, which could now be distinctly felt in the hypogastrium. By means of the counter pressure above the pubis, a much greater degree of pressure could be made upon the depression in the fundus of the uterus without lacerating its vaginal connections. At length the fingers of the left hand being pressed well down into the abdomen, seemed to fasten upon or hook over the anterior uterine lip and aid in its reflexion over the organ. Thus securely held between the two hands, one within the vagina and the other upon the hypogastrium, these efforts at reduction were continued until I became nearly exhausted from fatigue. Gradually the concavity of the fundus was found to be deeper and deeper, until it finally became completely restored. The bougie was now passed up to the fundus, penetrating twelve or more inches beyond the vulva, and gently maintained there by Prof. Hunt, whilst the patient was replaced in bed. My fingers were so benumbed by the long-continued unremitting pressure, as nearly to obliterate sensation, and at my request he also examined to ascertain whether the organs now occupied their normal relations. This being determined by him affirmatively, the bougie was gently withdrawn and the patient left with directions to give an anodyne, preserve quietude, and give her stimulants and nourishment freely. It may be added, that she seemed more comfortable than before the operation was undertaken, and expressed herself as feeling better than since her confinement. The hæmorrhage was, from this moment, completely arrested."

Two days afterwards the patient expired. On the succeeding day "the *post-mortem* was made by Dr. Lemon, in presence of Drs. Storck, Dupré, Hamestein, and Prof. Hunt, the last of whom, at my request, furnishes the following report of the condition of parts as they were found upon examination:—

'The examination was held eighteen hours after death. Only the abdominal cavity was opened. All the tissues were extremely bloodless. The stomach and intestines were fully inflated with gas, but held hardly any fluid contents.

The walls of the intestines were white and translucent, and no trace of inflammatory injection could be found, either upon them or any portion of the peritoneum. There was, however, a little serous effusion within the peritoneum, and between some of the convolutions of the intestine a very little lymph was exuded.

The uterus was dragged up and removed with as much of the vaginal cavity as could be reached from within. Externally the uterus presented its normal shape and position, there being no trace of its recent dislocation. The vaginal mucous membrane and the os uteri, presented the dark color usual to the organ at this period after labor. The tissues were not softened, nor was there any laceration of them at any point.

Upon section through the posterior wall the same pale, bloodless appearance, noticed elsewhere, was presented. The uterine cavity contained a little altered blood. Upon washing the surface it presented no unusual appearance. The situation of the placenta was marked by the usual rough, flocculent surface.

The examination revealed no cause of death, unless the anæmic condition of the tissues may be considered as such. I have never before seen so bloodless a subject, with one exception; that of a girl who died from purpura hæmorrhagica.'"

Unfortunate as was the result in this instance, the case, as Dr. White observes, will encourage practitioners to undertake reduction at a much later period than most writers have heretofore advised.

"Denman, Dewes, Velpeau, and others, believe any effort at restoration useless after a very few hours. In a valuable paper upon this subject from the editor of the Buffalo Medical Journal, to be found in the November number of that Journal for 1853, sixty-seven cases are collected, and all the facts pertaining to their reduction, so far as they could be obtained, are given. Most of the cases which were successfully treated were operated upon very soon after the accident. Thirty-two of the sixty-seven were not reduced, and a few 'exceptional cases' at various periods after the first day. By this table Dr. Hunt has shown that treatment has, though very rarely, resulted in success at this period than was formerly supposed practicable, and the above case furnishes another instance in support of the same position."

Dr. White concludes his article with the following :—

"The moderate anæsthesia, continued during the efforts of manipulation, doubtless saved the patient much pain and lessened involuntary resistance. Whether the patient's chances of rallying were improved by the reposition, may, by some, be deemed doubtful. There were no lesions of the utero-vaginal connection found, indicating that such a degree of force had been used as to impair the integrity of, or excite inflammation in those tissues. The hæmorrhage which had been considerable during the previous twenty-four hours, ceased with reduction, and the woman was much more comfortable the day following than the one preceding. The patient doubtless died from loss of blood immediately attending the delivery of the placenta and inversion of the uterus, the disturbance of the system occasioned by the unnatural position of that organ during eight days, and continuous drain by hæmorrhage during the same period. I believe it the opinion of all present that the shock of the operation of reposition was fully compensated by the increased comfort of the patient and arrest of flooding. She had, however, lost too large an amount of blood, reäction could not be established, though nature was aided in her efforts by all the resources of art."

Dr. Simpson in a paper published in the Edinburgh Medical and Surgical Journal, (see his Obstetric Memoirs, p. 724,) makes the following remarks upon the treatment of these cases :—

"In the *treatment* of inverted uterus, and in attempting that reduction of the organ which always forms our most immediate indication, Dr. Radford strongly advises us, provided the inversion is complete, and the plecenta still attached, to remove this latter mass, before we endeavor to compress and return the uterus itself. He has, as we have just hinted, fully shown that we need not be deterred from this practice by any dread of hæmorrhage. If the placenta, he observes, be completely detached from the uterus, this organ contracts as under ordinary circumstances, and that bleeding ceases. We need scarcely point out how much the separation of the placental mass will facilitate our attempts at reduction, and how highly necessary it is to effect this, if possible, before the inverted viscus becomes enlarged from congestion, and before the os uteri becomes too much irritated and contracted to admit of its easy return. In one case, Dr. Denman was unable to reduce the organ after the lapse of four hours. We must not, however, depair, if unsuccessful in our first efforts, or during the first stage. The inverted uterus has been reduced now in a number of instances, at a distance of several days from the occurrence of the accident, in cases where the threatened inflammatory symptoms had been kept in abeyance by the active measures that were adopted. Mr. Cawley, in one instance, reduced the organ after it had been down three days ; and in other cases Dr. Bradford reduced it after seven, and Mr. Ingleby after eight days. In one case Dr.

Belcombe succeeded, though the inversion had existed as long as twelve weeks. Cautious attempts of this kind, where there is no inflammatory complication, may, therefore, in some cases be warrantable, at a late period after the accident, with a view of saving the patient from those constant discharges, and other annoyances and miseries which the disease, when not remedied in its first stages, very generally entails."

It is a very singular, though a well attested fact, difficult as it is to account for it, that a spontaneous reduction of the inverted womb has been occasionally known to take place. Several cases of such an occurrence are related by Dr. Meigs in his Treatise on "Woman, her Diseases and Remedies," two of which fell under his own cognizance. They will be found described in his letter on Inversio Uteri, p. 241, 3d edition.—Ed.]

